# Yacon (*Smallanthus sonchifolius*) as a Food Supplement: Health-Promoting Benefits of Fructooligosaccharides

**DOI:** 10.3390/nu8070436

**Published:** 2016-07-21

**Authors:** Brunno F. R. Caetano, Nelci A. de Moura, Ana P. S. Almeida, Marcos C. Dias, Kátia Sivieri, Luís F. Barbisan

**Affiliations:** 1 Department of Morphology, Institute of Biosciences, Sao Paulo State University, Botucatu 18618-689, Brazil; brnncaetano@gmail.com (B.F.R.C.); nelcimoura@gmail.com (N.A.d.M.); 2 Departament of Food and Nutrition, Faculty of Pharmaceutical Sciences, Sao Paulo State University, Araraquara 14800-903, Brazil; anap.almeida@terra.com.br (A.P.S.A.); katiasiv@hotmail.com (K.S.); 3 Institute of Health Sciences, Federal University of Mato Grosso, Sinop 78550-000, Mato Grosso, Brazil; marcosdias16@gmail.com

**Keywords:** yacon, prebiotics, fructooligosacharides, functional food, chronic diseases

## Abstract

Yacon (*Smallanthus sonchifolius*), a perennial plant of the family Asteraceae native to the Andean regions of South America, is an abundant source of fructooligosaccharides (FOS). This comprehensive review of the literature addressed the role of yacon supplementation in promoting health and reducing the risk of chronic diseases. According to several preclinical and clinical trials, FOS intake favors the growth of health-promoting bacteria while reducing pathogenic bacteria populations. Moreover, the endproducts of FOS fermentation by the intestinal microbiota, short chain fatty acids (SCFA), act as substrates or signaling molecules in the regulation of the immune response, glucose homeostasis and lipid metabolism. As a result, glycemic levels, body weight and colon cancer risk can be reduced. Based on these findings, most studies reviewed concluded that due to their functional properties, yacon roots may be effectively used as a dietary supplement to prevent and treat chronic diseases.

## 1. Introduction

Yacon (*Smallanthus sonchifolius*) is a perennial herbaceous plant of the family Asteraceae, native to the Andean regions of South America [1,2]. This plant has a branching system that gives rise to aerial stems about 2 to 2.5 m high. Yacon yields starchy, fruit-like roots of different shapes and sizes that are usually consumed raw and taste sweet. Their crunchy texture very much resembles that of an apple. One plant is estimated to produce more than 10 kilos of roots [3,4]. The fact that the yacon plant adapts to different climatic regions, altitudes and soils explains its expansion outside the Andean region. Yacon is currently cultivated in Argentina, Bolívia, Brazil, the Czech Republic, Ecuador, Italy, Japan, Korea, New Zealand, Peru and the United States [4]. 

There is a variety of common names for yacon around the world. These include aricoma and aricuma in Bolivia, jicama, chicama and shicama in Ecuador, and arboloco in Colombia. However, the Spanish term yacon, derived from the Quéchua word “yaku” which means “watery”, is the most used worldwide. Interestingly, water is the most abundant component of the yacon root [2,4].

**Figure 1:**
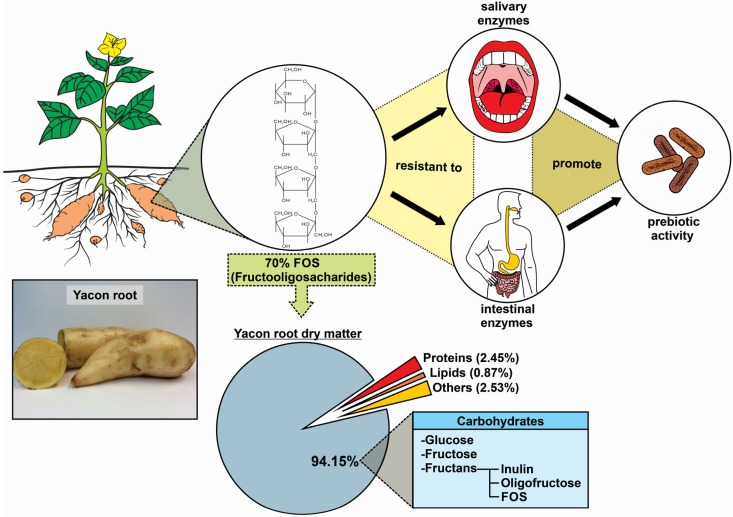
Chemical composition and functional properties of yacon roots.

Yacon roots’ water content usually exceeds 70% of the fresh weight while the major portion of the dry matter consists of fructooligosacharides (FOS) [5]. FOS content ranges from 6.4% to 70% of the dry matter (0.7% to 13.2% of the fresh weight) depending upon the specific crop and location. In yacon roots, the antioxidant capacity varies between 23 and 136 µmol/g trolox equivalent of the dry matter, and total phenolic compounds represent 0.79% to 3.08% of the dry matter [6,7,8]. Figure 1 summarizes the physicochemical and functional characteristics of yacon roots.

The high content of FOS in yacon roots is considered to offer health benefits, as it can reduce glycemic index, body weight and the risk of colon cancer [9]. Yacon functional properties, long recognized by folk medicine, have been the subject of a number of research projects and clinical trials [10]. Thus, the nutraceutical potential of yacon roots has garnered great public interest as a dietary supplement. In this comprehensive review, we focused on yacon FOS health-promoting benefits regarding human chronic diseases.

## 2. Fructooligosacharides: Bioactivity and Potential Health Benefits

Fructooligosacharides (FOS) are fructans consisting of linear short chains of fructose molecules. Fructans are synthesized from sucrose in the cell vacuoles of plant leaves, stems and roots. They help protect against drying out and are carbohydrate reserves in a wide number of plant families [11,12]. FOS are natural food components that can be found in garlic, onion, asparagus, artichoke, banana, wheat and yacon. However, the highest concentrations of FOS are found in yacon [13].

**Figure 2:**
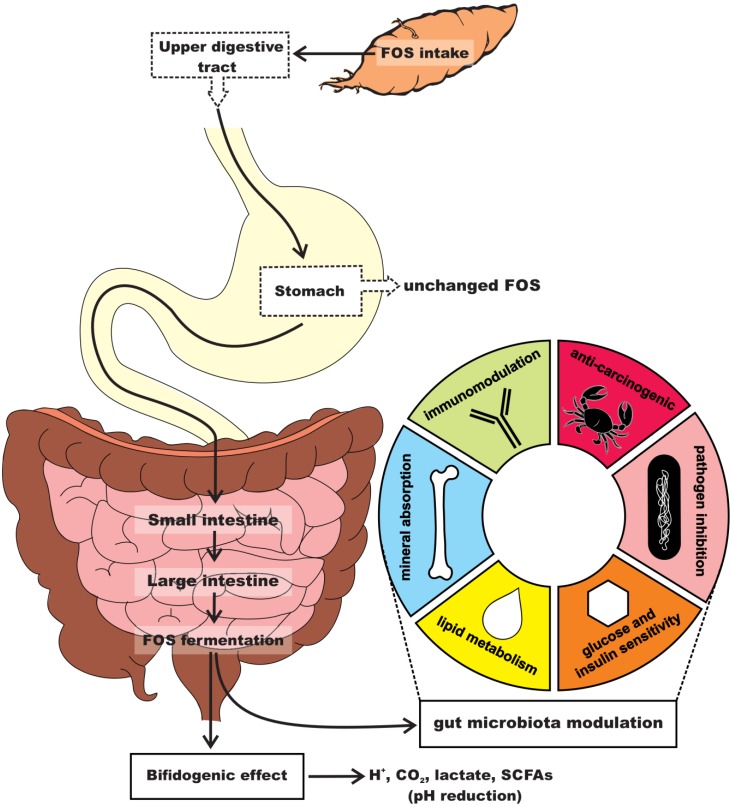
Yacon root consumption and health-promoting benefits of FOS.

FOS are able to escape enzymatic digestion in the upper gastrointestinal tract, reaching the colon intact before undergoing microbial fermentation. FOS intake elicits a bifidogenic effect by selectively stimulating the proliferation of bifidobacteria, a group of beneficial bacteria naturally found in the human colon (Figure 2) [14,15,16]. Short chain fatty acids (SCFA), the endproducts of FOS fermentation by the intestinal microbiota, can also favor the growth of health-promoting bacteria such as *Bifidobacterium* spp. and *Lactobacillus* spp., while reducing or maintaining pathogenic populations (e.g., *Clostridium* spp. and *Escherichia coli*) at low levels [17,18,19]. Thus, FOS are small soluble dietary fibers that exhibit prebiotic activity.

The term prebiotic was coined by Gibson and Roberfroid in 1995 to describe a “non-digestible food ingredient that beneficially affects the host by selectively stimulating the growth and/or activity of one or a limited number of bacteria in the colon, thus improving host health” [20]. This concept was later revised by Roberfroid who redefined a prebiotic as “a selectively fermented ingredient that allows specific changes, both in the composition and/or activity in the gastrointestinal microflora that confers benefits upon host well-being and health” [21].

Several other concepts have been proposed since then, but they all describe a prebiotic as a non-digestible compound able to selectively stimulate the growth of gut bacteria. According to the criteria proposed by FAO at the technical meeting on prebiotics [22], to be classified as a prebiotic, a compound must present the following qualifications: (a) component: a compound or substance that can be chemically characterized—not an organism or drug normally presented as a food-grade component; (b) health benefit: a compound or substance must resist digestion and absorption in the small intestine, over-riding any adverse effects; and (c) modulation: a compound or substance must promote health-related changes in the composition and/or activities of the colonic microbiota in the target host.

There is sufficient evidence to support the categorization of FOS as prebiotics. FOS offers physiological benefits that justify its use as a food supplement, particularly in cases of chronic diseases [14,23,24]. Since yacon has long been used in folk medicine for treating diabetes, constipation and various other human diseases, the present study aimed at reviewing the mechanisms underlying yacon FOS health benefits in colon cancer, diabetes, and obesity.

## 3. FOS Effects on Colorectal Cancer

Colorectal Cancer (CRC) is the third most commonly diagnosed type of cancer and a leading cause of death in the Western world. Although family history is an important risk factor for CRC development, only 15% of new cases have been linked to hereditary causes. In fact, the majority of CRCs (80%) occur sporadically and are associated with acquired risk factors, such as lifestyle and diet [25,26]. Dietary factors that potentially increase the risk of CRC include a high intake of red and processed meat, saturated fats and refined starches [27,28]. Diabetes and obesity are also associated with a higher risk of developing CRC [29].

Little is known about the feasibility, safety and efficacy of using dietary yacon to modulate or suppress CRC. Our research group was the first to report the chemopreventive effects of yacon root intake on dimethylhydrazine (DMH)-induced colon cancer in male rats. We showed a reduction in cell proliferation, number and multiplicity of preneoplastic lesions and invasive adenocarcinomas in a group receiving 1% of yacon powder [30]. In a more recent study evaluating the effects of yacon aqueous extract on the initiation step of CRC carcinogenesis, we found that yacon aqueous extract alone or that associated with *Lactobacillus acidophilus* (synbiotic formulation) reduced DMH-induced DNA damage in leukocytes. Moreover, we observed a reduction in cell proliferation indexes and a decrease in apoptosis levels in the group supplemented with the synbiotic formulation [31].

There is growing evidence that human intestinal microbiota plays an essential role in CRC carcinogenesis. The interplay between the intestinal microbiota, the intestinal epithelium and the host innate immune system is associated with several human diseases, including colitis and CRC [32,33]. Dysbiosis is a condition in which an imbalance in the microbial community favors the growth of specific pathogens that are potentially pro-carcinogenic. Intestinal microbiota disruption also exerts a great impact on colon metabolic profiles under the influence of the microbial community [34].

The influence of dietary habits on the composition of the microbiota has been widely accepted in the scientific community, supporting the hypothesis that diet patterns can induce dysbiosis [35]. Hence, the yacon root is thought to be a good dietary supplement, since its high content of FOS can selectively modulate the composition and function of the intestinal microbiota. FOS promote the growth of bifidobacteria, a genus of Gram-positive pleomorphic rods that play a regulatory role in the colon by inhibiting the growth of putrefactive bacteria. Bifidobacteria have been suggested to decrease the expression of xenobiotic-metabolizing enzymes and stimulate the immune system in the colonic mucosa [36,37,38].

FOS consumption also leads to increased SCFA production, primarily acetate, propionate and butyrate. Recent findings suggest that SCFA can suppress inflammation and cancer by increasing local immune response, decreasing colon pH and promoting ammonia and amine excretion [36,38]. During carcinogenesis, SCFA production in the colon by beneficial bacteria decreases cellular proliferation and induces apoptosis, especially in colon tumor cells. In fact, increasing butyrate production has also been shown to decrease the development of preneoplastic aberrant crypt foci lesions and delay tumor progression in rats [30,39,40].

FOS can indirectly influence immune activity via SCFA production that modifies the intestinal microbiota composition. SCFA promote a state of immune tolerance and modulate interleukin (IL) production and natural killer (NK) cell activity [41]. Vaz-Tostes et al. [42] reported that the consumption of yacon flour (0.14 g FOS kg body weight) over 18 weeks increased serum IL-4 and fecal secretory IgA in overweight preschool children with an inadequate dietary intake of zinc and fiber [42]. However, the role of prebiotic-induced immunomodulation in CRC is still unclear.

Increasing evidence suggests that FOS can also directly modulate the immune system through the gut-associated lymphoid tissue (GALT) rather than the gut microbiota [43]. Natural plant compounds such as fructans and polysaccharides may activate specialized immune cells (macrophages, dendritic cells, lymphocytes and neutrophils) by mimicking pathogen-associated molecular patterns (PAMPs) that bind to toll-like receptors (TLR), causing immunomodulatory effects [44]. For instance, TLR-mediated activation of NK cells can promote IFN-γ production and thus increase anti-tumor cytotoxicity. Furthermore, direct and indirect immunomodulation mechanisms can synergistically induce robust regulatory cellular immune responses [45]. Indeed, yacon treatment increased cytokine production (i.e., IL-10, IFN-α and IL-4) and the expression of toll-like receptor 4 (TLR4) and CD206 in cells in infant mice [46,47]. The increase in the expression of these receptors in gut-associated immune cells results in an enhanced status of the innate immune response with remarkable macrophage activity. The increased phagocytic activity of macrophages, mediated by the CD206 receptor and TLR4, is able to maintain colonic homeostasis without inducing inflammatory responses, reinforcing the intestinal barrier against pathogens and improving anti-tumor defense [47,48].

Table 1 shows that the effects of yacon consumption on colorectal cancer include: (a) suppressed cell proliferation; (b) reduced preneoplastic lesions; (c) significantly changed composition of the colonic microbiota; and (d) modulated immune response in CRC.

## 4. FOS Effects on Diabetes

Diabetes is the most common chronic disorder in developed countries, and a leading cause of death worldwide, with the global prevalence being 8.4% among adults (>18 years) in 2014 [49]. Obesity and physical inactivity have been related to increased risk of developing diabetes. Diabetes mellitus is a group of metabolic diseases characterized by hyperglycemia resulting from defects in insulin secretion and/or insulin action. Untreated chronic hyperglycemia can cause long-term tissue damage and dysfunction that might lead to adverse outcomes such as skin ulcers and amputations. Type 2 diabetes mellitus, characterized by insulin resistance and pancreatic β-cell dysfunction, is the most common form of diabetes [50,51].

The current standard care for diabetes type 2 prevention and management is dietary intervention [52]. Hence, antidiabetic nutraceuticals, such as yacon, with reduced or no side effects have been high in demand. Due to their hypoglycemic properties, yacon roots have long been recognized by folk medicine as an effective alternative for diabetes treatment. Moreover, yacon roots, either crude or refined, can be used as low-calorie sweeteners by dieters as well as people suffering from diabetes [53].

Several preclinical and clinical trials have shown that yacon root FOS have a notable hypoglycemic effect. In an experiment using streptozotocin-induced diabetic rats, the number of insulin-positive pancreatic cells and glucagon-like peptide-1 (GLP-1) significantly increased, while visceral abdominal fat was reduced and fasting insulin serum levels were slightly increased in diabetic rats supplemented with yacon flour (340 or 6800 mg FOS/kg body weight (bw).) for 90 days [54]. In another study using Zucker fa/fa male rats, yacon at 6.5% in chow reduced blood glucose levels and improved hepatic insulin sensitivity. In this case, dietary yacon significantly reduced Trb3 hepatic expression and increased Akt expression, improving insulin sensitivity in the liver [55].

In a trial evaluating the daily intake of freeze-dried yacon among elderly individuals, FOS content (7.4 g) was positively correlated with decreasing serum glucose levels [56]. Among obese and slightly dyslipidemic pre-menopausal women, Genta et al. observed that yacon syrup at 0.14 g/kg bw reduced fasting serum insulin and was significantly associated with decreased beta-cell function and insulin resistance in a homeostasis model assessment (HOMA), suggesting that yacon syrup FOS promote glucose absorption in peripheral tissues and improve insulin sensitivity via SCFA production [57].

Plasma glucose homeostasis is achieved through a tightly controlled balance between glucose input (food intake and liver production) and glucose uptake by multiple organs [58]. FOS putative effects on glucose disposal and insulin tolerance are mediated via multiple mechanisms. These mechanisms are part of the milieu of interactions that take place between the intestinal microflora and the host metabolism, and converge to a similar outcome—the production of SCFA by FOS fermentation. SCFA produced by the intestinal microbiota are promptly absorbed in the colon and conveyed into blood, where they play their physiological roles as substrates or signaling molecules [59,60,61].

Several studies have been conducted to elucidate the underlying mechanisms of SCFA on glucose homeostasis. For instance, acetate has been shown to reduce free fatty acids (FFA) plasma levels, which are known to cause peripheral insulin resistance in obese individuals, inhibiting glucose uptake and glycogen synthesis [62]. The oral administration of propionate to both diabetic hyperglycemic and normal rats has been shown to decrease gluconeogenesis by increasing AMPK expression in the liver [63]. SCFA have also been reported to affect glycemic levels through the gut hormones peptide YY (PYY) and GLP-1 by directly activating colonic free fatty acid receptors 2 and 3 (Ffar2 and Ffar3). PYY and GLP-1 have also been proposed to improve plasma glucose levels after a meal in a dependent manner, stimulating insulin and inhibiting glucagon secretion in the pancreas [64,65].

Table 2 shows that the effects of yacon consumption on diabetes include: (a) increased glucose absorption in peripheral tissues; (b) decreased gluconeogenesis; (c) improved insulin tolerance in the liver; and (d) increased insulin secretion in the pancreas.

## 5. FOS Effects on Obesity

Overweight and obesity comprises one of the main public health challenges worldwide because of the associated increased risk of developing type 2 diabetes, heart disease, hypertension, cancer and a number of other diseases [66]. Over the past few decades, the increasing number of overweight and obese people has been claimed as a pandemic. According to the World Health Organization (WHO), the prevalence of overweight was estimated to be 39% among adults aged 18 years and over, while obesity represented 13% of the overall world’s adult population in 2014 [67]. Overweight and obesity are defined as a condition of abnormal or excessive accumulation of adipose tissue in the body. This condition may impair health and lead, for instance, to the development of chronic inflammation and metabolic syndrome [68]. The main causes of overweight and obesity are related to energy imbalance (i.e., energy intake exceeds energy expenditure) modulated by metabolic factors, diet and physical activity. Hence, there has been a global trend to an increasing intake of energy-dense foods that are rich in saturated fat and refined starches, as well as increasing rates of physical inactivity and a sedentary lifestyle [69].

Metabolic syndrome is a cluster of cardiometabolic risk factors that arises from insulin resistance accompanying abnormal visceral adiposity, glucose intolerance, dyslipidemia and hypertension [70]. As a consequence, metabolic syndrome leads to a state of chronic inflammation produced by a complex interaction between genetic and environmental factors. At the moment, there is no consensus on what is the most appropriate nutritional intervention for treating metabolic syndrome related to obesity [71]. However, certain dietary bioactive compounds found in over 800 plants can help to prevent or ameliorate multiple facets of metabolic syndrome. In this regard, yacon has been hypothesized to exert anti-obesity and hypolipidemic effects by improving biochemical parameters and satiety [72]. Though there is a popular claim that yacon syrup can aid in weight loss, scientific evidence is nevertheless scarce. These properties, however, are thought to be directly related to the high content of FOS found in yacon root.

In a sub-chronic four-month oral toxicity study, dried yacon root (340 mg and 6800 mg FOS/kg bw) was given as a diet supplement to healthy, non-obese Wistar rats. During the feeding trial, yacon administration was well tolerated and did not produce any toxic effect. Furthermore, yacon consumption at both doses significantly reduced post-prandial serum triacylglycerol (TAG) levels [73]. Similar findings were reported when yacon flour (340 or 6800 mg FOS/kg bw) was administered to streptozotocin-induced diabetic rats. The oral consumption of yacon flour decreased fasting plasma TAG, very low-density lipoprotein (VLDL) and the postprandial peak of plasma TAG [54]. In another study using synbiotic formulations, a positive effect on TAG and high-density lipoprotein (HDL) cholesterol levels was reported in diabetic rats that received an aqueous extract of yacon roots and soybean, in association or not with *Enterococcus faecium* CRL 183 and *Lactobacillus helveticus* ssp jugurti [74].

Although the hypolipidemic effects of yacon roots have been demonstrated in pre-clinical studies, evidence from well-designed human trials is still scarce. As cited before, in a study with premenopausal, obese and slightly dyslipidemic women, yacon syrup intake (0.14 g FOS/kg bw) over 120 days showed improvements in fasting low density lipoproteins (LDL) and visceral fat [57]. Otherwise, no such effect was reported in a study conducted in elderly who consumed a daily intake of freeze-dried powdered yacon [56]. Moreover, yacon administered to healthy individuals (6.4 g FOS/day) over two weeks markedly accelerated colonic transit in a placebo-controlled, double-blind study design [75].

The beneficial effects of FOS on lipid metabolism are well recognized, although the underlying mechanisms are still unclear. FOS exert hypolipidemic effects through SCFA production by the intestinal microbiota, resulting in the modulation of biochemical and cellular pathways related to lipid metabolism, satiety and intestinal transit [76]. Indeed, SCFA have been shown to positively regulate the lipid homeostasis by inhibiting lipolysis, increasing triglyceride mobilization and adipogenic differentiation [60,77]. In vitro studies also reported that SCFA were able to reduce cholesterol synthesis by decreasing hepatic activity of the 3-hydroxy-3-methylglutaryl-CoA synthase (HMGCS) and 3-hydroxy-3-methylglutaryl-CoA reductase (HMGCR) enzymes [68]. AMPK activation by SFCAs has also been suggested to inhibit HMGCS and HMGCR activation in an independent manner [60].

It has also been shown that dietary FOS are able to increase the secretion of peptides by the gastrointestinal diffuse neuroendocrine system via SCFA production, acting as modulators of appetite and increasing satiety [78]. The physiological control of satiety is partly regulated by intestinal peptide secretion including cholecystokinin (CCK), PYY and GLP-1. It is noteworthy that this regulation is complex and involves a range of mechanisms and multiple control systems [79]. Nevertheless, SCFA can directly increase PYY and GLP-1 secretion by Ffar1 and Ffar2 activation in the colon [80]. Conversely, long-term studies have suggested that a long exposure time is needed for the intestinal microbiota to adapt and produce the amounts of SCFA to elicit the physiological effect of satiety. Increased gut motility may also be affected by intestinal peptide secretion [81]. However, SCFA such as butyrate are able to exert direct effects on myenteric neurons and increase the intestinal motility, supporting the hypothesis by which a high fiber intake accelerates the colonic transit [82].

Although there have been several studies reporting the beneficial effects of yacon intake on obesity, much needs to be understood about the mechanisms and processes that underlie such effects. Table 3 shows that the effects of yacon consumption on obesity include: (a) modulated biochemical and cellular pathways related to lipid homeostasis; (b) increased satiety; and (c) increased gut motility.

## 6. Yacon Consumption Adverse Effects

Although yacon consumption is safe at recommended dosages, overdosing may be uncomfortable, but not life-threatening. Symptoms of yacon overdose include abdominal pain, bloating, flatulence and diarrhea [57]. In addition, yacon consumption markedly accelerates colonic transit, increasing stool frequency [75]. The only report of adverse effects found in the literature describes the case of a 55-year-old woman who developed anaphylaxis after yacon ingestion [83].

A side effect that should be taken into account when evaluating the proportion of oligofructans/fructose within yacon roots is the partial hydrolysis of yacon oligofructans to fructose that starts shortly after harvest and may accelerate during food processing [84]. This can seriously affect yacon’s health-promoting benefits because high-fructose administration correlates with the induction of insulin resistance by modifying the early steps of insulin signal transduction [85]. Therefore, cold storage and temperature-controlled environments are highly recommended to keep the functional properties of the yacon roots [84].

## 7. Conclusions

Experimental and clinical studies have reported that yacon consumption is important to regulate several pathways related to colon cancer, diabetes, and obesity. The FOS content found in yacon roots can modulate the human intestinal microbiota, increase glucose absorption in peripheral tissues, stimulate insulin secretion in the pancreas and modulate cellular pathways related to lipid homeostasis. Therefore, based on these findings, most studies reviewed concluded that due to their functional properties, yacon roots may be effectively used as a dietary supplement to prevent and treat chronic diseases.

## Figures and Tables

**Table 1:** Effects of yacon consumption on colorectal cancer.

Yacon Source	Research, Subject Randomized, Dose and Duration	Health Properties	References
Dried extract of yacon root	Mouse (BALB/c) Dose: 340 mg/kg day in diet, for 75 days	Growth of *Bifidobacteria* and *Lactobacilli*	Bonet et al. [16]
Dried extract of yacon root	Rats (Wistar) Dose: 0.5%, 1.0% (20.4% FOS) in diet for 13 weeks	Reduce tumor multiplicity, preneoplastic lesions and cell proliferation	De Moura et al. [27]
Aqueous extract of yacon root	Rats (Wistar) Dose: 2.2 mL (1% FOS) for 8 months	Reduce DNA damage and cell proliferation	Almeida et al. [28]
Dried extract of yacon root	Mouse (BALB/c) Dose: 3.0%, 5% FOS in diet for 30 days	Improves the immune parameters	Delgado et al. [46]

**Table 2:** Effects of yacon consumption on diabetes.

Yacon Source	Research, Subject Randomized, Dose and Duration	Health Properties	References
Yacon flour	Rats (Wistar)	Increase insulin-positive pancreatic cell	Habib et al. [54]
	Dose: Yacon flour (340 mg FOS/kg/day) for 90 days		
Dried extract of yacon root	Rats (Zucker fa/fa)	Improve insulin sensitivity in the insulin-resistant state	Satoh et al. [55]
	Dose: 6.5% yacon for 5 weeks		
Dried extract of yacon root	Elderly man and woman	Decrease in serum glucose levels	Scheid et al. [56]
	Dose: Yacon powder (7.4 g of FOS) for 9 weeks		
Yacon syrup	Obese and slightly dyslipidemic pre-menopausal women	Improve insulin-resistance state	Genta et al. [57]
	Dose: Yacon syrup (0.29 g and 0.14 g FOS/kg/day), for 120 days		

**Table 3:** Effects of yacon consumption on obesity.

Yacon Source	Research, Subject Randomized, Dose and Duration	Health properties	References
Yacon flour	Rats (Wistar) Dose: Yacon flour (340 mg FOS/kg/day) for 90 days	Hypolipidemic effect	Habib et al. [54]
Yacon syrup	Obese and slightly dyslipidemic pre-menopausal women Dose: Yacon syrup (0.29/g and 0.14/g FOS/kg/day) for 120 days.	Increased defecation frequency and satiety sensation	Genta et al. [57]
Dried extract of yacon root	Rats (wistar) Dose: Dried yacon root (340 mg and 6800 mg FOS/bw) for 4 months	Reduced post-prandial serum TAG levels	Genta et al. [73]
Aqueous extract of yacon root	Rats (wistar) Dose: 1 mL/kg body weight/day, 4.30 g/100 g of frutans, for 7 weeks	Positive effect on TAG and HDL	Roselino et al. [74]
Yacon syrup	Healthy individuals Dose: 6.4 g FOS/day	Accelerates the colonic transit	Geyer et al. [75]

## Abbreviations

The following abbreviations are used in this manuscript:

BW	body weight
CRC	colorectal cancer
CCK	cholecystokinin
DMH	1,2-dimethylhydrazine
GLP-1	glucagon-like peptide 1
SCFA	short chain fatty acids
FFA	free fatty acids
FFAR	free fatty acid receptor
FOS	fructooligosacharides
HDL	high density lipoprotein
LDL	low density lipoprotein
PAMPs	pathogen-associated molecular patterns
PYY	peptide YY
TAG	tryacylglycerol
TLR	toll-like receptors
VLDL	very low density lipoprotein
